# Comparing event-related potentials of retrospective and prospective metacognitive judgments during episodic and semantic memory

**DOI:** 10.1038/s41598-023-28595-z

**Published:** 2023-02-02

**Authors:** Metehan Irak, Can Soylu, Mustafa Yavuz

**Affiliations:** 1 Department of Psychology Brain and Cognition Research Laboratory, Bahçeşehir University, Çırağan Cad. No: 4 Beşiktaş, Istanbul, 34353 Turkey; 2 Graduate School of Systemic Neurosciences, Ludwig-Maximilians-Universität München, Munich, Germany

**Keywords:** Psychology, Cognitive neuroscience

## Abstract

It is unclear whether metacognitive judgments are made on the basis of domain-generality or domain-specificity. In the current study, we compared both behavioral and event-related potential (ERP) correlates of retrospective (retrospective confidence judgments: RCJs), and prospective (feeling of knowing: FOK) metacognitive judgments during episodic and semantic memory tasks in 82 participants. Behavioral results indicated that FOK judgments reflect a domain-specific process, while RCJ reflect a domain-general process. RCJ and FOK judgments produced similar ERP waveforms within the memory tasks, but with different temporal dynamics; thus supporting the hypothesis that retrospective and prospective metacognitive judgments are distinct processes. Our ERP results also suggest that metacognitive judgments are linked to distributed neural substrates, rather than purely frontal lobe functioning. Furthermore, the role of intra-subject and inter-subject differences in metacognitive judgments across and within the memory tasks are highlighted.

## Introduction

Metacognition is defined as “cognition about cognition”, “knowing about knowing”, or the ability to reflect on, monitor and control cognitive processes^1,2^. Whether metacognitive judgments are a domain-general process (a common process employed independent of task type) or a domain-specific process (where different processes are involved depending on the type of task)^3^, is a controversial topic. Metacognitive judgments can be retrospective (e.g. retrospective confidence Judgments [RCJs] and judgment of learning [JOL]) or prospective (e.g. feeling of knowing [FOK] and ease-of-learning [EOL]). The other controversial issue in metacognition literature is whether the domain characteristics of metacognitive judgments are affected or determined by when the metacognitive judgment is made (retrospective or prospective) and the type of memory task (semantic or episodic). In this study, we examined RCJ and FOK during episodic and semantic memory tasks to clarify these issues by combining event-related potentials (ERPs) and behavioral data from the same sample.

FOK is a metacognitive process which allows individuals to make predictions about their likelihood of remembering in the future any information they currently cannot recall, based on whether they feel like the information is stored in their memory^4–7^ (e.g. a student may predict their success on an upcoming exam by reflecting on their current level of knowledge). However, RCJ is the subjective evaluation of one’s level of confidence in a previous decision (after recall). RCJ is a type of metacognitive process allowing self-monitoring of performance^8^ (e.g. after the exam, a student may then estimate their grade without feedback). Metacognitive judgments can be named based on the type of task measured. For example, in this study, the RCJ and FOK judgments were measured during semantic and episodic memory tasks. Semantic-FOK refers to FOK judgments (FOKs) measured duringsemantic memory tasks. The sematic-FOK relates to recall of general knowledge or facts that were learned before the experiment (e.g. “What is the capital of USA?”), while episodic-FOK relates to recall of newly learned information (e.g. word-pairs) within a particular context^4^. RCJs on the other hand, are obtained after retrieval of each item, with participants being asked to rate their levels of confidence in their answers. It is suggested that FOK is based on a sense of familiarity with the cue^9,10^ or on the amount and accessibility of partial information retrieved in relation to the target or both (heuristics), whereas RCJs are thought to be related to the strength of the memory trace^11–13^.

The episodic-FOK is assumed to rely on autonoetic consciousness, reflecting the retrieval of partial information from the learning phase. For example, an episodic-FOK judgment may be based on an individual’s ability to recall what he/she was thinking when first presented with the stimuli. Semantic-FOK is based on the retrieval of specific semantic knowledge^8,14,15^. Semantic-FOK and episodic-FOK are hypothesized to rely on distinct retrieval processes^16^, which supports the domain-specific view of metacognition. Previous studies (e.g.^17–20^), which used episodic memory (learning word-pairs) or semantic memory (e.g. knowing general knowledge questions) tasks, supported the domain-specific view hypothesis with evidence for impaired episodic-FOK but preserved semantic-FOK in older adults and patients with Alzheimer’s disease^17^, frontal lobe lesions^18^, and schizophrenia^19,20^. However, other studies which also used different experimental tasks (e.g. visual memory and perceptual task) have found evidence for domain-generalizable processes in metacognition^3,21–25^. There are two plausible explanations for this discrepancy. The first is the variability ofexperimental tasks, and the second is the variability of recognition procedures (e.g. yes/no; two/four alternative-forced choices) that were used in different studies.

While FOK concerns the prediction of future performanceFOK judgments are made after a retrieval attempt and are presumed to share the characteristics of both prospective and retrospective judgments^26^. However, RCJ is the subjective evaluation of past retrieval success. Therefore, RCJs may offer insight into the domain-generalizability of metacognitive judgments.

### The neurobiology of metacognitive judgments

Neuroimaging studies^5,18,27–30^ have shown that medial prefrontal cortex (PFC) activation is associated with prospective metacognitive judgments, however rostrolateral-PFC activity correlates with RCJs suggesting that these are two distinct processes. In addition, fMRI studies indicate that several brain regions are differentially associated with semantic and episodic metacognitive judgments^31–34^. ERP studies (e.g.^35–38^) using various tasks (e.g. math problems, face-name recognition, episodic memory, learning related and unrelated word pairs) reported that FOKs elicited P200^35,36^, N200^37^ and P300^35^, while JOL linked to P200^36,37^, N200^37^ and N400^38^ peaks. These studies concluded that FOK and JOLs are associated with different ERP components, and these ERPs are linked to perceptual fluency and conflict processes, which supports the familiarity-based model^36,37^. It was also proposed that^38^ processing fluency and explicit beliefs about memory contribute to JOL. These ERPs were obtained from the frontal, central and parietal areas, which affirmed that metacognitive judgments appear to be linked to distributed neural substrates. The N400 was also recorded during low EOL response, whereas high EOL produced slow-wave activity, suggesting N400 is linked to encoding fluency cues, while slow-wave is associated with metacognitive monitoring^39^. Also a mid-frontal fN400-like response were recorded during the Dunning-Kruger Effect Judgment. Researchers concluded over- and under-estimators use different cognitive processes (familiarity and recollection, respectively) when making their judgments^40^.

Compared to FOK, the nature of RCJ is negligible. Furthermore, the ERP correlates of the retrospective memory judgments are often studied during recognitionrather than recall tasks and recognition (or memory) confidence. These studies consistently reported that the frontal N400 relates to a familiarity (know); however, the later parietal old/new component (LPC) associates with a recollection process (remember) (e.g.^41–48^). Additionally, ERP correlates of recollection and familiarity are affected by the level of memory confidence (e.g.^49–52^). However, it was concluded that memory confidence is not the primary determinant of the remember/know distinction, and these two memory judgments result from qualitatively different memory processes^41^. Recently^53,54^, reported small ERP changes on the FN400 and LPC, suggesting that decision criterion must be considered when interpreting ERPs of recognition memory.

### The goal of the study

It is still debated whether metacognition is based on a global resource that is applied to different tasks or if self-evaluative processes are task-specific. It has been shown that domain-specific and domain-general neural signals co-exist in the human brain^55^. However, their interplay may differ according to the type of memory tasks and metacognitive judgments. Although behavioral studies have provided evidence for differences between retrospective and prospective metacognitive judgments and their accuracy, the ERP correlates of these judgments have received less attention. Individuals make metacognitive judgments based on both experience-based and information-based cues, although the reliance on each varies depending on judgment type^56^. Thus, it is possible that these metacognitive judgments differ across tasks. Thus, our first hypothesis is that correlations between semantic-RCJ and episodic-RCJ and correlations between semantic-FOK and episodic-FOK will not be significant. It was previously shown that^26^ RCJ and FOK are distinct processes and FOKs are thought to be domain-specific and RCJs to be domain-general. We also hypothesized that correlations between semantic-RCJ and semantic-FOK, and between episodic-RCJ and episodic-FOK will not be significant.

Individuals with high metacognitive performance in one modality are likely to perform well in other modalities^57^ andpeople who are overconfident in one cognitive task also tend to be overconfident in other tasks, too^21,58^. We compared ERPs elicited by RCJ and FOKs during episodic and semantic memory tasks in the same sample. We chose RCJ (retrospective) and FOK (prospective) metacognitive judgments. Although episodic metacognitive judgments are associated with ERP peaks obtained in the early time window (200 ms after stimulus onset), to the best of our knowledge, no study examines semantic metacognitive judgments. Besides, previous studies have shown that semantic memory is associated with ERPs recorded in the middle (300–500 ms) and late (400–800 ms) time windows. Thus, following previous studies (e.g.^8,21,26,59^) we expected a significant main effect of memory type and we secondly hypothesized that episodic-FOK and episodic-RCJ would be related to the enhanced amplitude of the early ERP components (around 200 ms), however semantic-FOK and semantic-RCJ would be related to the enhanced amplitude of the middle ERP components (around 300–400 ms). In addition, parallel with our first hypothesis, we also expected significant main effects of judgment types in which the ERP amplitudes of RCJ and FOK would be different.

## Results

### Statistical analysis plan

First, to test our first hypothesis, correct recognition, correct recall, gamma correlations, da, meta-da, metacognitive bias and metacognitive efficiency scores were compared using a 2 (task type: episodic and semantic) × 2 (judgment type: FOK and RCJ) ANOVA. Then, the correlations between behavioral variables were tested. Second, to test our second hypothesis, and to compare mean ERP amplitudes of RCJ and FOK during two memory tasks, we employed a mass univariate approach using non-parametric cluster-based permutation tests. Then, statistical analyses were performed in two designatedsignificant time windows, using 2 (judgment type: RCJs and FOK) × 3 (hemisphere: left, right and midline) × 3 (region: frontal, fronto-central and parietal) repeated measure ANOVAs.

### Behavioral results

#### *First-order, d,* and meta-*d performance*

To test our first hypothesis, the task performance was evaluated in terms of the proportion of correct recall and recognition, and Type 1 *d′* performance. The correlations between *d*_*a*_ and meta-*d*_*a*_ for episodic-FOK (*r* = 0.29), semantic-FOK (*r* = 0.30) and semantic-RCJs (*r* = 0.26) were significant (all *p* < 0.01), but not for episodic-RCJs. Although, the differences between RCJ and FOK and differences between the episodic and semantic memory were not significant, the difference between semantic (*M* = 30.89) and episodic (*M* = 36.40) mean percentage of recall performance for *d*_*a*_ was significant (*F*(1,81) = 6.48, *p* < 0.01). Similarly, percentage of episodic recognition (*M* = 88.51) was higher than semantic (*M* = 42.96) recognition (*F*(1,81) = 104.56, *p* < 0.001). There was no significant correlation between semantic and episodic recall.

#### Metacognitive bias

**Figure 1:**
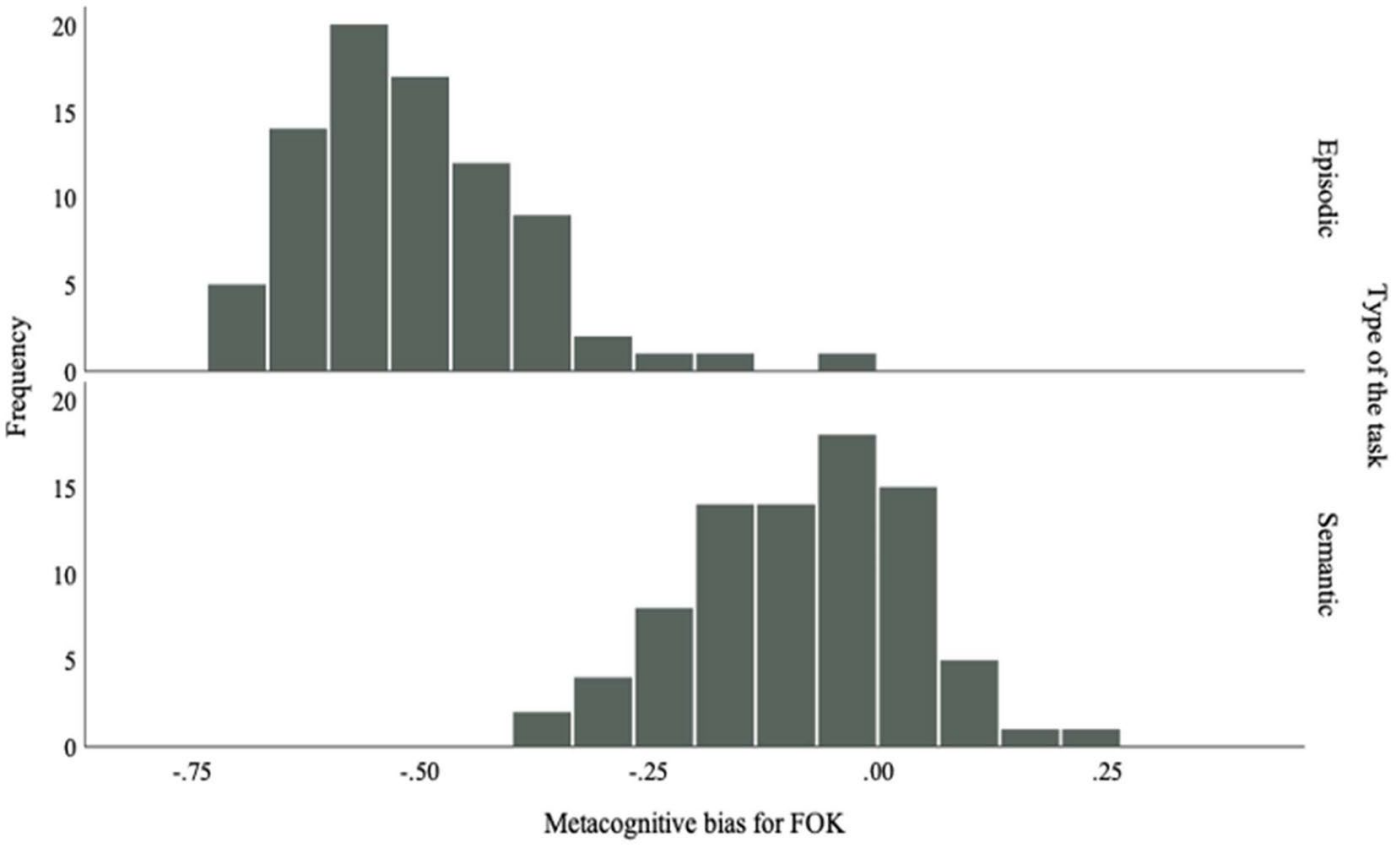
Metacognitive sensitivity during FOK phases of episodic and semantic memory tasks.

**Figure 2:**
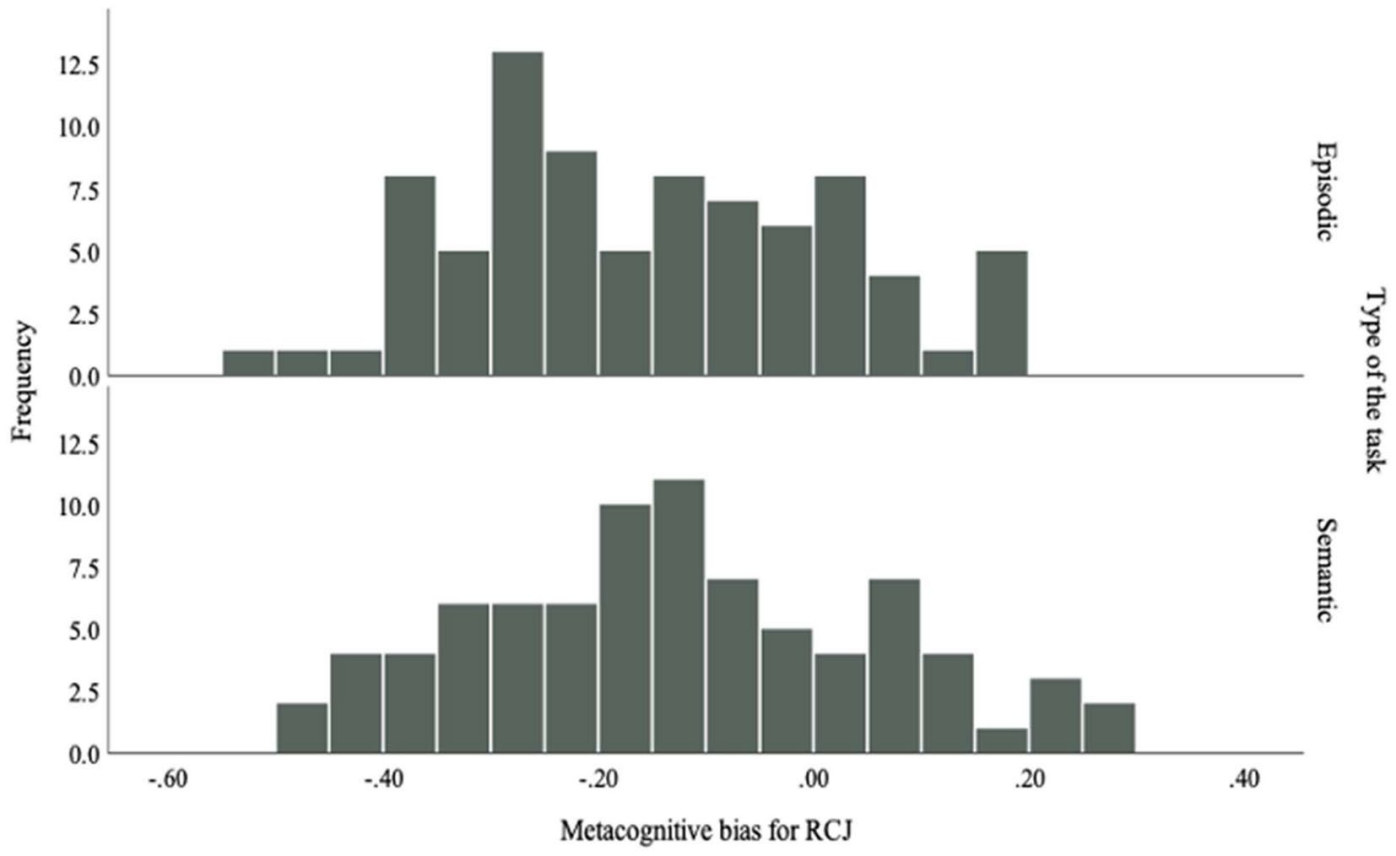
Metacognitive sensitivity during RCJ phases of episodic and semantic memory tasks.

Metacognitive bias (e.g. the tendency to be overconfident—positive value—or underconfident—negative value—^60^) was calculated (mean confidence minus mean performance) for the RCJ and FOK phases of each task (Figs. 1 and 2). Results showed significant main effect of task (*F*(1,324) = 138.15, *p* < 0.001, *η*^*2*^ = 0.30, *d*_*z*_ = 0.21), with higher *d*_*z*_ values in the semantic memory (*M* = 0.16, *d*_*z*_ = 0.89) than the episodic memory task (*M* = − 0.05, *d*_*z*_ = − 0.20). There was also a significant main effect of judgment type, *F*(1,324) = 144.40, *p* < 0.001), with higher values for RCJs (*M* = 0.16, *d*_*z*_ = 0.90) than FOK judgments (*M* = − 0.06, *d*_*z*_ = − 0.22). The interaction between judgment and task type was significant, *F*(1,324) = 126.29, *p* < 0.001, *d*_*z*_ = 0.21. Metacognitive bias scores were lower for episodic-FOK (*M* = − 0.27) than semantic-FOK (*M* = 0.15), and higher for semantic-RCJ (*M* = 0.17) than episodic-RCJ (*M* = 0.16). The difference in metacognitive bias scores between the memory tasks was larger for FOK judgments than RCJs. The correlation between semantic-RCJ and episodic-RCJ metacognitive bias scores was significant (*r* = 0.26, *p* < 0.05), but not significant between episodic-FOK and semantic-FOK.

#### Metacognitive efficiency

Metacognitive efficiency (meta-*d*_*a*_/*d*_*a*_ ratio) is a subject's level of metacognitive sensitivity, given a certain level of task performance. Metacognitive efficiency quantifies the level of metacognitive sensitivity relative to first-order performance^61,62^. ANOVA results showed that the mean meta-*d*_*a*_/*d*_*a*_ ratio value was 49% accuracy for episodic-FOK, 25% accuracy for semantic-FOK judgments (*p* > 0.05). For RCJ, it was 73% for episodic-RCJ and 56% for semantic-RCJ (*p* > 0.05). Differences in gamma correlation, meta-*d*_*a*_ and *d*_*a*_ values and correlations between metacognitive efficiency scores for either memory and judgment type were not significant.

#### Correlational analyses among behavioral variables

The correlations between semantic- recall and the semantic- meta-*d*_*a*_/*d*_*a*_ ratio (*r* = 0.30, *p* < 0.01); between semantic-FOK and episodic-FOK *d*_*a*_ (*r* = 0.36, *p* < 0.01) and between episodic-RCJ and semantic-RCJ *d*_*a*_ (*r* = 0.26, *p* < 0.05) was significant, but not significant between the semantic-meta-*d*_*a*_/*d*_*a*_ ratio and episodic-recall. Semantic-FOK and episodic-FOK sensitivity (meta-*d*_*a*_) was not correlated with semantic-recall and episodic-recall. FOKs are made immediately following the RCJs. Thus, FOKs may be more biased towards target accessibility than cue familiarity. Correlations between RCJs and FOKs were examined to investigate this possible bias, however, results were insignificant (*r* ≤ 0.13).

### ERP results

#### Mass univariate analysis results

**Figure 3:**
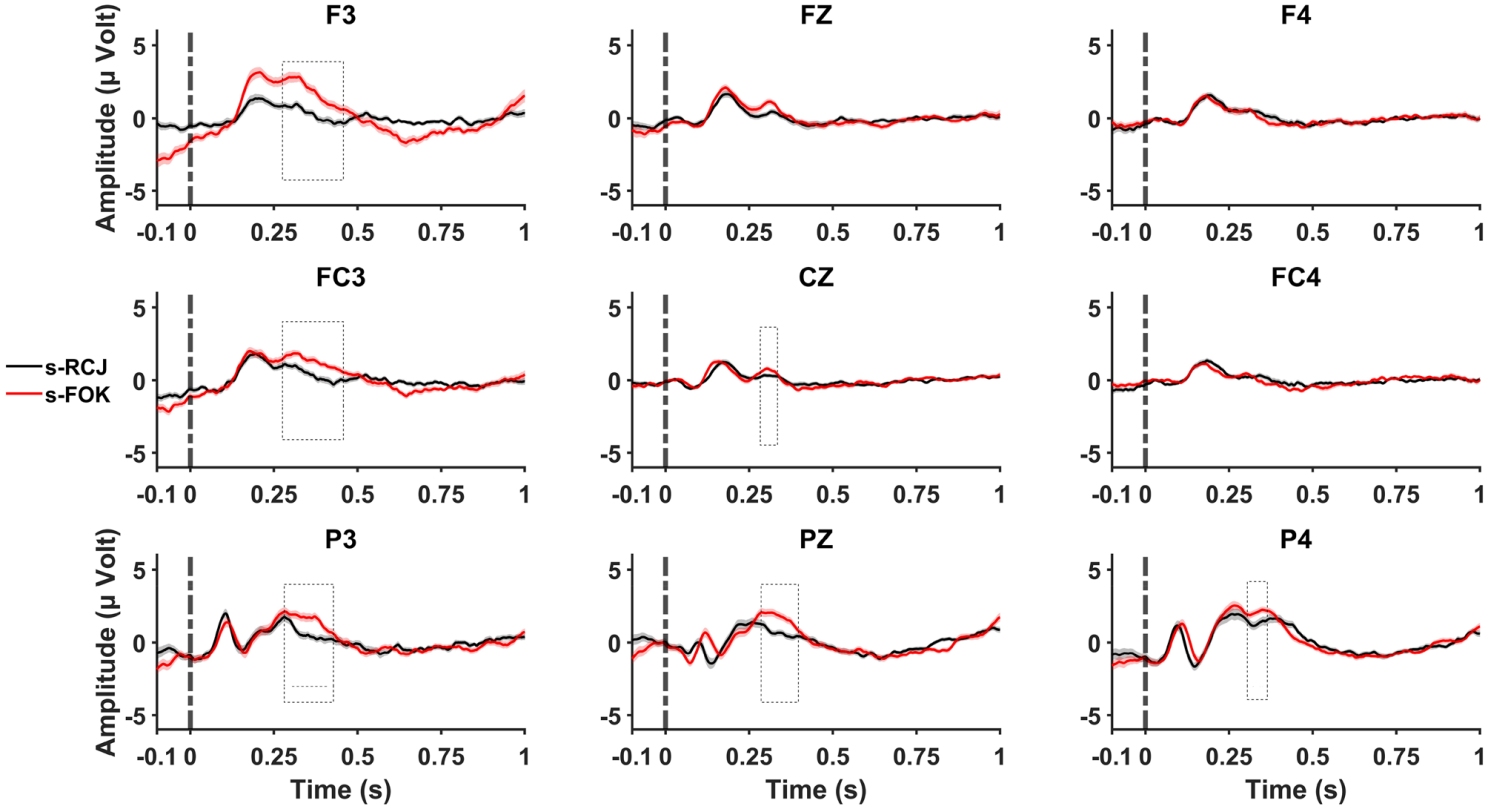
Stimulus-locked ERP grand average with standard errors during the semantic memory task for RCJ (black line) and FOK (red line) at F3, Fz, F4, FC3, Cz, FC4, P3, Pz, and P4 electrode sites Stimulation applied at “0.0 ms” time point (stimulus onset). Cluster-based permutation tests were run within 50–900 ms, and dashed boxes indicate latencies that were found to exhibit significant effects.

**Figure 4:**
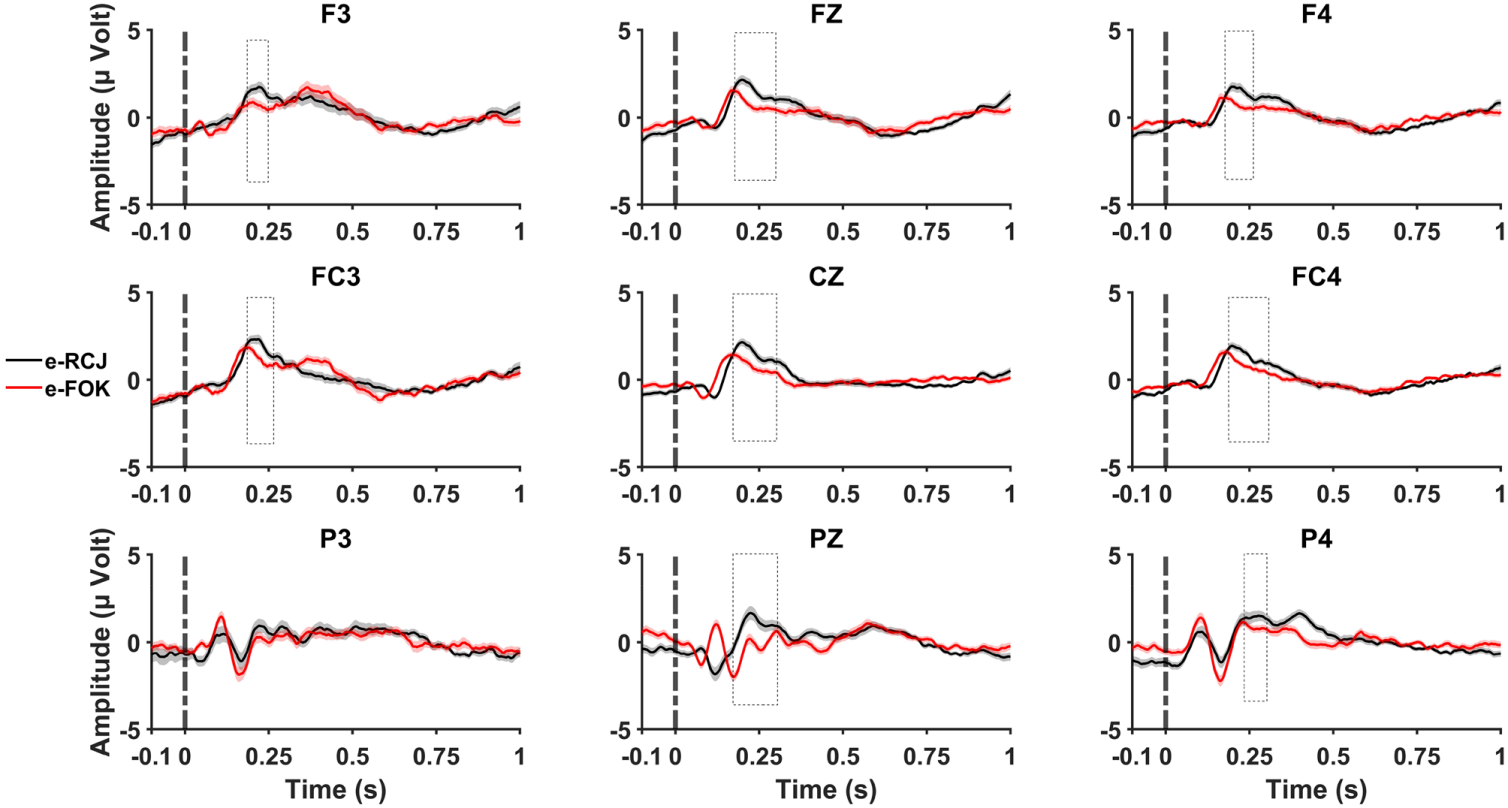
Stimulus-locked ERP grand average with standard errors during the episodic memory task for RCJ (black line) and FOK (red line) at F3, Fz, F4, FC3, Cz, FC4, P3, Pz, and P4 electrode sites Stimulation applied at “0.0 ms” time point (stimulus onset). Cluster-based permutation tests were run within 50–900 ms, and dashed boxes indicate latencies that were found to exhibit significant effects.

**Figure 5:**
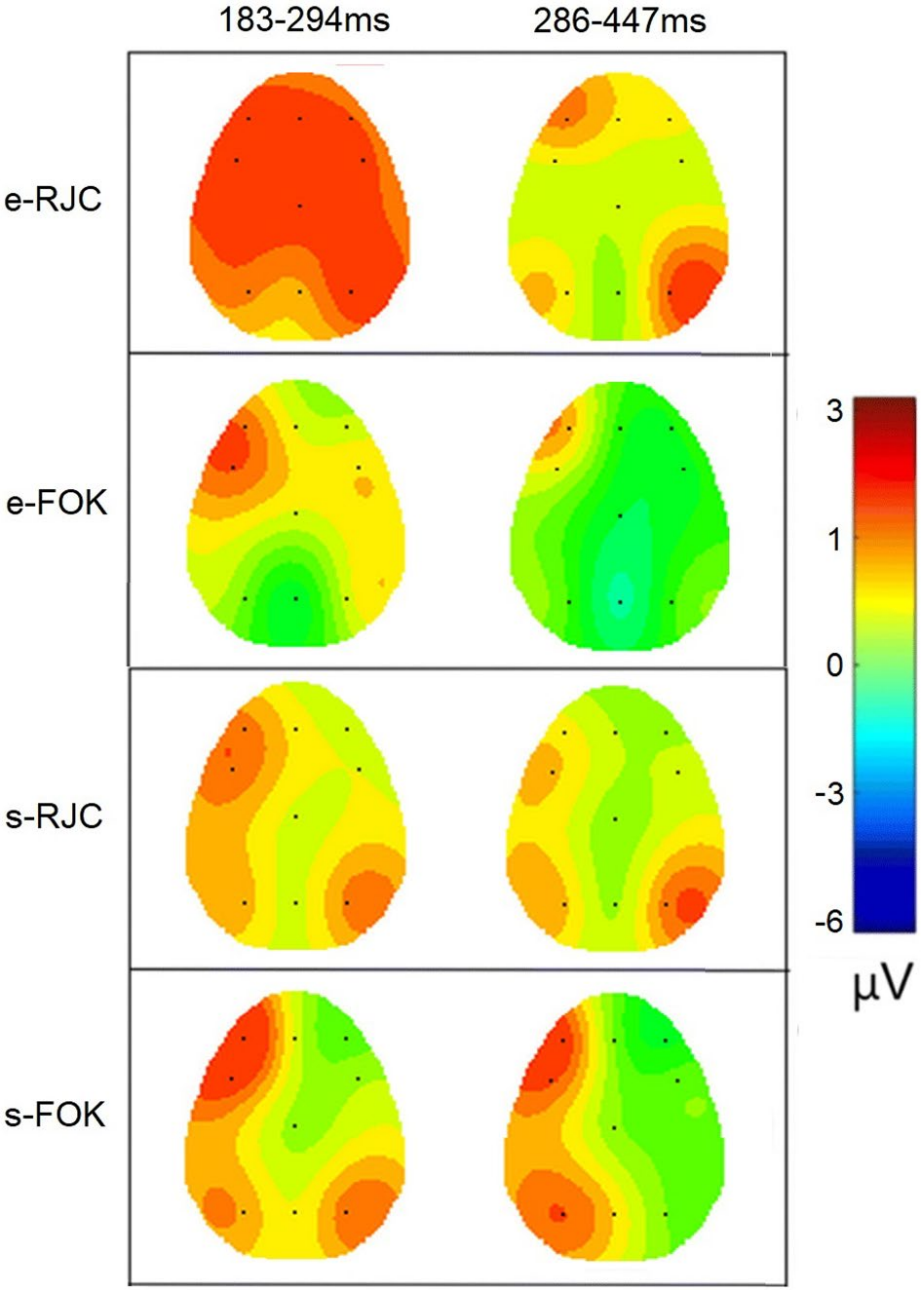
Head map of the cluster-based permutation tests that were conducted between 50 and 900 ms during episodic (top) and semantic (bottom) memory tasks for RCJ (top) and FOK (bottom) decisions. For each task and decision, the topographic distribution was plotted in two significant time windows, 183–294 ms (left column) and 286–447 ms (right column).

Figures 3 and 4 illustrate the ERPs triggered in response to metacognitive judgments for each memory task, respectively. On the other hand, Fig. 5 shows the topographic distribution plotted in two significant time windows for each task and each metacognitive judgment. To test the second hypothesis, cluster-based permutation statistics were conducted on each memory task to examine and determine specific time samples with significant differences in ERP amplitudes across judgment types. This first-level analysis revealed significant time clusters, which further constrained our analysis reported in the following sections.

For the semantic memory task, the test revealed a significant cluster (*p* = 0.004) between 280 and 450 ms, this difference was observed at the FC3, F3, Cz, P3, Pz, and P4 electrodes. The cluster statistics (calculated via the maximum sum of observed t-values for the significant cluster) was -1.836 (*df* = 99). However, for the episodic memory task, the test revealed a significant cluster (*p* = 0.008) at 183–294 ms, this difference was observed at all electrodes except P3. The cluster statistics for the observed group difference was 1.487 (*df* = 99) (Figs. 3 and 4).

Following these, statistical analyses were performed using mean voltage amplitudes of ERP waveforms in two designated time windows, using 2 × 3 × 3 repeated measure ANOVAs that included Greenhouse–Geisser corrections in cases where factors had more than two levels. All reported differences were significant at the level of *p* < 0.05 and were followed by Bonferroni tests (α = 0.05), where necessary. Mean and standard errors are presented in Table 1.

**Table 1:** Mean and standard error according to memory task, judgment type, region and hemisphere.

Judgment type	Region	Hemisphere	Semantic memory		Episodic memory	
			*M*	*SE*	*M*	*SE*
RCJ	Frontal	Midline	0.12	0.19	1.46	0.21
		Right	0.12	0.18	1.34	0.21
		Left	0.20	0.10	1.25	0.24
	Fronto-central	Midline	0.17	0.10	1.46	0.21
		Right	0.21	0.15	1.41	0.16
		Left	0.36	0.20	1.70	0.22
	Parietal	Midline	0.47	0.16	1.03	0.32
		Right	1.37	0.25	1.04	0.30
		Left	0.51	0.20	0.54	0.33
FOK	Frontal	Midline	0.24	0.16	0.73	0.18
		Right	0.24	0.11	0.68	0.16
		Left	1.85	0.28	0.73	0.19
	Fronto-central	Midline	0.22	0.12	0.76	0.17
		Right	0.16	0.11	0.88	0.13
		Left	1.38	0.17	1.12	0.18
	Parietal	Midline	1.30	0.13	0.23	0.19
		Right	1.64	0.19	0.60	0.18
		Left	1.43	0.22	0.19	0.23

#### Semantic memory task

Main effects of judgment type (*F*(1, 66.14) = 27.74, *η*_*p*_^*2*^ = 0.36), region (*F*(1.17, 56.45) = 12.26, *η*_*p*_^*2*^ = 0.20), and hemisphere (*F*(1.37, 66.16) = 16.95, *η*_*p*_^*2*^ = 0.26) were significant (all *p* < 0.001). The FOKs were higher in amplitude than the RCJs. Higher amplitudes were observed at the parietal than at the fronto-central and frontal regions. Also, the amplitudes at the left hemisphere electrodes were higher than those at the central and right hemisphere electrodes.

Two way interaction effects between judgment type and hemisphere (*F*(1.63, 78.56) = 47.27, *η*_*p*_^*2*^ = 0.50), and between region and hemisphere (*F*(2.67, 128.19) = 22.82, *η*_*p*_^*2*^ = 0.32), and a three way interaction effect between judgment type, region, and hemisphere (*F*(2.93, 140.85) = 8.99, *η*_*p*_^*2*^ = 0.16) were significant (all *p* < 0.001). Amplitudes for FOKs were higher than for RCJs in the left hemisphere at frontal and central regions. In the right hemisphere, higher amplitudes were observed at the parietal regions than at the fronto-central and frontal regions.

#### Episodic memory task

Main effects of judgment type (*F*(1, 78.03) = 33.26, *η*_*p*_^*2*^ = 0.41), and region (*F*(1.23, 59.45) = 6.75; *η*_*p*_^*2*^ = 0.12) were significant (all *p* < 0.001). RCJs were higher in amplitude than FOKs. Higher amplitudes were observed at the fronto-central than the parietal region. The interactions between judgment type and hemisphere (*F*(1.66, 80.07) = 4.16, *η*_*p*_^*2*^ = 0.08), region and hemisphere (*F*(2.22, 107.00) = 4.64, *η*_*p*_^*2*^ = 0.09), and judgment type, region, and hemisphere (*F*(2.57, 123.46) = 4.30, *η*_*p*_^*2*^ = 0.08) were significant (all *p* < 0.01). RCJs were higher in amplitude than FOKs in the left hemisphere and central. RCJs were higher in amplitude than FOKs on the right side. In the left hemisphere, higher amplitudes were observed at the fronto-central than the parietal region. For central electrodes, lower amplitudes were observed at the parietal region than the fronto-central and frontal. Within the frontal region, RCJs produced higher amplitudes than FOK on the left side, centrally, and on the right side. Within the fronto-central region, RCJs produced higher amplitudes than FOK on the left side, centrally, and on the right side. Within the parietal region, RCJs produced higher amplitudes than FOK at central electrodes.

## Discussion

### Behavioral results

Our results support previous studies^21,59,61,63^, showing that efficiency for FOK is lower than for RCJs and that the overlap between semantic-RCJ and episodic-RCJ is higher than that between FOKs. In line with previous results (e.g.^26,59,62^), we found that cross-task correlations between recall and recognition and within-task correlations between metacognitive judgments were not significant, also metacognitive judgments are more consistent with memory performance during episodic memory compared to semantic memory task.

The efficiency scores were better for RCJ compared to FOK, suggesting that RCJ is more consistent with actual memory performance than FOK. Also, metacognitive efficiency was higher for episodic-FOK than semantic-FOK, supporting the hypothesis that FOK differs across tasks. Parallel with previous studies^16,64,65^ during episodic-FOK, but not semantic-FOK, participants can use partially retrieved information as a cue. This further supports the suggestion^16,31,64–66^ that episodic recall employs autonoetic consciousness, allowing one to re-experience the remembered information, whereas semantic recall is based on the accessibility of semantic information associated with the specific question.

During episodic-FOK if someone is able to recall full or partial information about a word pair recently learned during the encoding phase, the episodic-FOK reflects an attempt to make an accurate judgment. Thus similar processes may underlie RCJ^26^. On the other hand, semantic-FOK is based on attempts to recall general knowledge that were learned previously. People make high FOKs when their search for an answer to a question brings to mind a large amount of partial information that is also intense and vivid. Thus, individuals make semantic-FOKs based on the activation of a network of related information^67^. For instance, if someone does not know the answer to the general knowledge question, it is still possible to make a semantic-FOKs, but the accuracy may be lower. Consistent with a recent study^26^, although cross-task correlation for FOK metacognitive efficiency was not significant, it was significant for RCJ, suggesting that FOKs reflect a domain-specific while RCJs reflect a domain-general process.

Retrospective and prospective metacognitive judgments have been found to be uncorrelated and associated with activation of different brain areas, suggesting that these two metacognitive judgments were different^5,16,28,68–70^. People make FOKs about their future memory performance. In this case, people have insight into, and receive feedback regarding their own performance. However, RCJ is the estimation of previous recall ability without feedback. Therefore, although it is reasonable to assume that FOK partially relates to retrospective recall judgments, since RCJs and FOKs were uncorrelated in our study, we concluded that different retrieval processes underpin these two metacognitive judgments. Although both retrospective and prospective judgments are strongly related to the strength of memory performance^5,71,72^, the evaluative processes underlying these judgments differ. For instance, while FOK is based on familiarity^73,74^, accessibility^2,11,67,75,76^ or both—heuristics—^15,77–79^, JOL and RCJs mainly relate to the accessibility of the cue^13,77,80^. Retrieval is a combination of several factors such as memory trace, sources, current goal and the test query^80–83^. Thus, these processes may also affect metacognitive judgments.

### N200, P200, and metacognition

N200 is related to different process (e.g. inhibition, conflict, monitoring, perceptual novelty, attentional deviation, and cognitive control)^35,36,84–87^. While it is not possible to locate the neural generators of ERPs from their scalp distributions alone, comparing our ERP findings with previous fMRI studies may give an idea, albeit limited. The neural organizations of prospective (e.g.^33,34,88–90^) and retrospective memory (e.g.^62,70,91^) are different. Consistent with these, we recorded posterior N200 and its amplitude varied across memory task and judgment type. Also, significant differences were found during the episodic memory task at the Pz electrode. Supporting previous studies, we concluded that the posterior N200 is related to the monitoring and control processes during metacognitive judgments and that posterior areas also contribute to metacognitive processes.

The P200 has been found to be related to different cognitive functions, including working and short-term memory^92^, the similarity effect, coding, rehearsal^93^ and perceptual processing^35^. Previous ERP studies^35–37^ recorded P200 response during FOK judgments have concluded that FOK judgments are based on perceptual fluency and conflict processes which in turn are used to guide metacognitive judgments. Accordingly, we concluded that P200 has two functions during metacognitive judgments: first, assessment of stimulus familiarity, and second, search/control and monitoring. In other words, during FOK and RCJs, individuals make a rapid assessment of the familiarity and identity of the stimulus in order to initiate effective and successful search and monitoring.

### P300 and metacognition

The P300 has been found to be related to various memory processes (e.g. activation of memory representation, updating, confidence, decision formation, and familiarity^94–100^. P300 amplitude has previously been shown to be affected by the degree of FOK^36^, with higher FOK producing larger amplitudes than low FOK. fMRI studies showed that different brain regions are involved in varying confidence levels (e.g.^68,70,101^). Also confidence and decision information-seeking choices were similarly modulated by P300 during a perceptual decision task, and it was concluded that these neural markers of confidence reflect information-seeking decisions^102^. Thus, we concluded that the P300 we obtained during metacognitive judgments is linked to activation of memory representations and memory updating of previously evaluated stimuli. After the familiarity analysis, a search and monitoring process occurs at around the 200 ms time window, yielding the N200 and P200 responses, then, memory representations and memory updating occur for familiar stimuli at the next time interval, yielding the P300 response.

We showed that although semantic-FOK produced larger amplitudes than semantic-RJC, episodic-RCJs had larger amplitudes than episodic-FOK. The observed amplitude differences between RCJ and FOKs may not provide sufficient evidence that they are distinct processes. However, we found non-significant correlations between RCJ and FOKs. Thus, our ERP and behavioral results supported our hypotheses that RCJ and FOKs are distinct processes and the results reflect different underlying retrieval processes during these judgments.

Uncertainty might negatively affect cognitive processes, meaning that emotional processes become more influential during decision making; thus confidence judgments about decisions may be inconsistent. Furthermore, uncertainty may lead to misrepresentation of the correct response^103,104^. Uncertainty increased P200 amplitudes, while certainty increased later ERPs, including P300, N400, and LPC^105,106^. During the semantic memory task, if an individual does not know the answer to a question, retrieving even partial information about the answer would be very difficult. This may lead to reduced semantic-RCJ amplitude since the process is somewhat all-or-nothing (high certainty). On the other hand, during semantic FOKs, uncertainty might be low since participants knew that they would be given hints in the future. This may explain the larger amplitudes during FOKs than RCJs.

Our memory tasks consisted of different types of stimuli and metacognitive judgments are task sensitive. Our results also support previous literature, which showed variations in ERP components based on the types of memory tasks and types of stimuli and differences in the ERP components of metacognitive judgments measured within these distinct tasks^4,35–37^. Thus, relationships between ERPs and metacognitive judgments should also be investigated using different memory tasks.

### Relationships between ERPs and behavioral outcomes of metacognitive judgments

Metacognitive bias score indicates the difference between participants’ metacognitive judgments and their objective accuracy levels. During the Dunning-Kruger Effect task^40^, a type of retrospective metacognitive judgment, the over-estimators responded faster than under-estimators when their estimate was in the highest percentile, and they responded more slowly when heir estimate was in the lowest percentile. We found that participants were overconfident during RCJ while underconfident during FOKs; also they were overconfident during semantic memory while underconfident during episodic memory tasks. Our participants tended to give higher and more accurate RCJs than FOKs. Lastly, although the accuracy of RCJs was higher in episodic memory, FOKs were more accurate in semantic memory task. Consistent with previous work (e.g.^21,23,26,61^), we found a significant correlation between semantic-RCJ and episodic-RCJ metacognitive bias scores, but not between episodic-FOK and semantic-FOK. Thus, these results also supported that retrospective and prospective metacognitive judgments are distinct processes while RCJs are domain-general, but FOKs are domain-specific. Also, these differences are consistent across the memory tasks, which showed significant condition effect. We also concluded that external feedback might affect bias scores. For example, participants made RCJs without receiving external feedback about their previous recall, while they made FOKs after feedback about their future performance. Therefore, both the presence of feedback and making this estimation prospectively (for an indefinite period) may be one factor creating the bias difference between RCJ and FOK.

Following Rouault et al.^55^, we argue that there are important implications when using behavioral versus neurobiological data to investigate whether metacognitive judgments are domain-specific or domain-general. Although behavioral studies in healthy and neurological populations indicate that different resources underlie a mental process, this assumption has not been fully supported by neurobiological studies. We concluded that this is because domain-general processes may be affected by psychological (e.g. stress, worry, anxiety) and/or physiological factors (e.g. fatigue, age) that also affect domain-specific processes. As discussed earlier, studies suggest that domain-specific and domain-general neural signals co-exist in the human brain^55^ and are implicated in human and animal subjects’ fronto-parietal networks in metacognitive judgments (e.g.^31,107–112^). It has been proposed that metacognitive judgments depend on gradient neural circuits that are shared and spread within the brain^55^. While the strength of the relationship between these circuits and different types of metacognitive evaluations might vary, the type of task could also have an effect. Thus, we argue that it is possible to observe intra-subject variations in metacognitive judgments across tasks and intra-subject differences in metacognitive judgments within the task.

There are some limitations to the present study. We used standard experimental tasks and procedures to measure metacognitive judgments. However, using a 6-point Likert-type scale to assess metacognitive judgments may not be optimal since the decision criterion affects recognition judgments and ERPs^53,54^ and the type of decision criterion varied across the studies (e.g.^33,34,36,37^). Secondly, in line with previous studies, there was no time limit for some phase of the experiment, but decision time may be a confounding variable for neuroimaging studies^113,114^. Thirdly, there are different types of episodic and semantic memory tasks and metacognitive judgments have been also shown to be task-sensitive (e.g.^2,11,14,36,37,67,76^). Lastly, while FOK judgments were given only for unrecalled items due to the nature of the tasks, RCJs were given for all items. Future studies should address these issues.

In conclusion, our results provided direct evidence for distinct neurobiological correlates that may reflect metacognitive processing during episodic and semantic FOK and RCJs. These judgments produced different behavioral and temporal dynamics, supporting the hypothesis that retrospective and prospective metacognitive judgments are distinct processes. We also observed that intra-subject and inter-subject variations play an important role during metacognitive judgments.

## Methods

### Participants

Eighty-five undergraduate students from several departments participated in this study. All were right-handed and reported normal or corrected-to-normal vision. Participants with neurological and/or psychiatric disorders and those who were taking or had recently stopped taking antidepressants, psychotherapeutic drugs, or drugs that could affect cognitive functions were excluded. Three participants were excluded due to equipment failure or excessive electroencephalographic (EEG) artifacts. The remaining 82 participants (51 female) had a mean age of 21.18 (range 18–26).

### Experimental tasks

#### Semantic memory task

**Figure 6:**
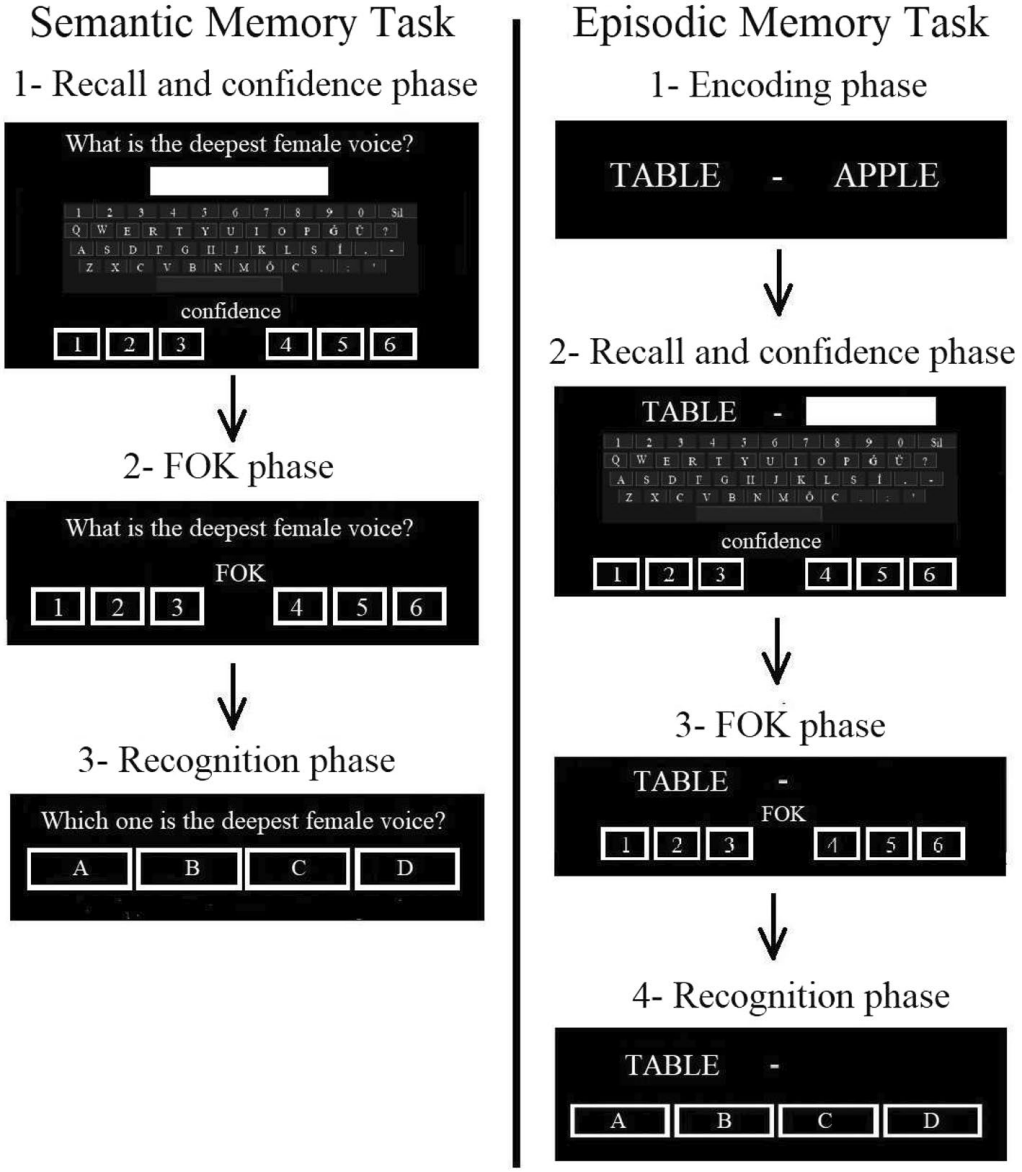
Semantic and episodic memory tasks were used in the study.

Thirty general knowledge questions (e.g., What is the deepest female voice? see Fig. 6) were selected from the norming study^115^. The questions with the highest frequency were equally selected in each category (easy, medium, and difficult), and the word length of the questions ranged between 29 and 96. The classical recall-judgment-recognition paradigm was used^7^. Participants were instructed to perform each phase of the task as fast and accurately as possible. In the first (recall) phase, participants were asked to answer general knowledge questions. Participants gave their answers using the on-screen digital keyboard during the recall phase for both tasks. Participants were also instructed to write “no” for the answers they did not know or remember and indicate the confidence rating as one. These answers were considered incorrect and were presented during the FOK judgment phase. However, these were not included in the calculations of the RCJ mean. Typing errors (e.g., ‘Grece ‘instead of’ Greece’; ‘conputer’ instead of ‘computer’) in the answers were accepted as correct. After each answer, participants were instructed to provide their level of confidence for recall (RCJ) using a 6-point Likert-type rating scale (1: definitely not sure, 6: *definitely sure).* Participants were also given a definition and example of RCJ. In the FOK phase, the participants were asked to make a FOK judgment for the items they failed to remember. They were given the definition and an example of a FOK judgment and then made their judgment based on the following question: "Even though I don’t remember the answer now, would I be able to pick the correct answer from among the several choices in the future?”, using a 6-point Likert-type rating scale (1: I will definitely not be able to find the correct answer, 6: I will definitely be able find the correct answer). The participants then completed a recognition test, in which each of the failed questions was presented once again with the multiple choice options featuring the correct answer and incorrect alternatives. The alternative options for each question were the same as in the original study.

#### Episodic memory task

176 words were selected from a Turkish word-frequency database^116^ to be used as task stimuli (Fig. 6). All were high frequency nouns, having five or six letters. Eighty-eight of the words were used to make up a study list of 44 word-pairs. The remaining words were used in the recognition phase of the task. To eliminate semantic relatedness, the word-pairs were created by random matching. To minimize primacy and recency effects, the first and last two word-pairs from each list were not included in the statistical analyses.

A classical recall-judgment-recognition paradigm (e.g.^7,28,91^) was used to measure FOK performance. The task consisted of four consecutive phases. In the first (learning) phase, each word-pair was presented and participants were instructed to memorize the words. In the second phase (cued-recall and RCJ), the first word of each word-pair was presented and participants were asked to recall which word it was paired with. Then after the response they were asked to indicate their level of confidence (RCJ) using a 6-point Likert-type rating scale (1: definitely not sure, 6: definitely sure*).* In the third phase they were asked to give a FOK judgment for the words they failed to remember. They were given the same instructions as in the semantic memory task. Participants then completed a recognition test in which each cue word was presented along with alternatives: the correct answer, one word from the study list, and two words from the initial pool.

RCJ and FOK accuracy (the ability to predict retrospective and prospective memory performance) was calculated for both tasks, separately, using the Goodman–Kruskal’s Gamma correlation, a commonly used measure of FOK accuracy^117^. In addition, *d*_*a*_ (the distance between the means of the SDs^118^, meta-*d*_*a*_ (a measure of metacognitive sensitivit^62,119,120^), and metacognitive bias (mean confidence minus mean performance; the standardized mean difference effect size for within-subjects designs is referred to as Cohen’s *dz*, where the *z* alludes to the fact that the unit of analysis is no longer X or Y, but their difference^23,60,121^) were calculated for each participant, for each task, and for both FOKs and RCJs. We used the original formulas from the studies we referred to for calculating these scores. For calculating these scores, we used the original formulas in the studies we have referred to.

### Procedure

Participants gave informed consent to participate in the study after the purpose and the nature of the experiment was fully explained. Participants were advised to abstain from lack of sleep, alcohol and caffeine on the evening before the study. The study conformed to the Declaration of Helsinki and was approved by the Bahçeşehir University Scientific Research and Publication Ethics Committee. Participants provided written informed consent and received financial compensation and/or course credits in return for their participation. Participants were informed that they were free to withdraw from the study at any point. The duration between each stimulus (after a decision was made) was 500 ms in all phases of the experiment. Except for the JOL and FOK phases, there was no time limit during any phase of the experiments. However, these two phases were presented in three seconds. The experiment was programmed using Visual Studio 2010 Ultimate on a Windows XP computer with a 21-in. monitor. Each stimulus was typed in black, Arial 24-point, uppercase letters on a white background. Eye to screen distance was approximately 75 cm. The order of the experimental tasks was counterbalanced. The full study session lasted approximately 2.5 h.

### EEG recording and preprocessing

ERPs were recorded while the participants made RCJs and FOK Judgments. EEG/EOG signals were recorded for 1200 ms after the stimulus onset using 32 Ag/AgCl electrodes (30 scalp EEG electrodes and 2 reference electrodes) mounted in elastic Quick-caps (Neuromedical Supplies, Compumedics, Inc., Charlotte). EOG signals were measured from two bipolar channels: one was formed by two electrodes placed at the outer canthus of each eye; another, by two electrodes below and above the left eye. EEG signals were recorded from 30 electrodes (FP1, FP2, F7, F8, F3, F4, Fz, FT7, FT8, FC3, FC4, FCz, T7, T8, C3, C4, Cz, TP7, TP8, CP3, CP4, CPz, P7, P8, P3, P4, Pz, O1, O2, Oz) arranged according to the standard 10–20 system, with two additional electrodes placed at BP1/BP2 and also on the left and right mastoids (M1/M2). All EEG electrodes were referenced on-line to an electrode at vertex and re-referenced off-line to linked mastoids. EEG and EOG signals were amplified and recorded at a 1000 Hz sampling rate using Synamp2 amplifier at AC mode (Neuroscan, Compumedics, Inc., Charlotte) with high- and low-pass filter set at 0.15 and 100 Hz, respectively. EEG electrode impedance was kept below 5 kΩ.

EEG data pre-processing was conducted using Edit 4.5 (Neuroscan, Compumedics, Inc. Charlotte) and applied to each participant’s dataset. Data were down-sampled to 250 Hz to reduce computational demands and then low-pass filtered at 30 Hz and high-pass filtered at 0.15 Hz. EEG segments were extracted with an interval of 100 ms preceding and 1000 ms following the stimulus onset. Artifact rejection was performed in two steps. First, trials containing activity exceeding a threshold of ± 100 µV at vertical and horizontal EOG and EEG channels were automatically detected and rejected. Second, we manually removed trials with saccades identified over the horizontal EOG channel and we also verified that this was done for each participant and each electrode For the computation of ERPs, artifact-free segments were baseline-corrected using a 100 ms pre-stimulus period and then averaged for the experimental conditions. ERPs were obtained by stimulus-locked (stimulus onset) averaging of the EEG recorded in RCJ and FOK judgment conditions.

### Data analysis

Averages of ERPs were determined in the temporal direction. In order to obtain overall averages, acquired and filtered epochs from 82 participants were derived according to the task phase and/or type of response. All participants who had at least 20 artifact-free trials per condition were included in the analyses (13 subjects were excluded). Thus, ERP data from 69 participants who gave RCJ and FOK judgment ratings for both semantic and episodic memory tasks were included in the calculation of ERP averages.

To compare mean ERP amplitudes of RCJ and FOK during episodic and semantic memory tasks, we employed a mass univariate approach using the FieldTrip Toolbox (version no: 20180306)^122^ running under Matlab R2017a (MathWorks, Natick, MA, U.S.A.). The comparisons for both memory types were conducted using non-parametric cluster-based permutation tests^123^. The epochs, which were stimulus onset for RCJ and FOK judgments phase of the episodic and semantic memory tasks were averaged over time for each participant. ERP amplitude differences between RCJ and FOKs were investigated in a within-memory type fashion. Based on previous neuroimaging literature on metacognitive judgments, discussed above, we focused on three electrode zones: frontal (F3/Fz/F4), fronto-central (FC3/Cz/FC4) and parietal (P3/Pz/P4). We detected extreme noise on the C3 and C4 electrodes due to equipment failure, thus these were excluded from the statistical analysis and FC3 and FC4 electrodes were included. Thus, nine pre-selected electrodes were included in the analysis (FC3, F3, FZ, F4, FC4, CZ, P3, PZ, and P4).

Non-parametric cluster-based permutation tests were conducted, based on participants' ERPs per each condition. Then, grand averages were computed solely for visualization purposes. Data were analyzed using the FieldTrip toolbox’s^122^ “*ft_timelockstatistics*” function. Correction for multiple comparisons was conducted using the temporal clustering (*correctm* = *“cluster”*) method. The pre-specified alpha value for univariate tests was 0.025 (two-tailed); t-tests were conducted using the 50–900 ms post-Judgment time interval. Clusters were quantified based on the maximum cumulative t-values (*clusterstatistic* = *“maxsum”*) with a pre-specified significance threshold of 0.05 (*clusteralpha*). The time-locked average epochs were resampled 1000 times between each condition.

### Statistical power analysis

A power analysis using the G*Power program^124^ indicated that to investigate effects of independent variables (judgment type, hemisphere, and region) on amplitude and latency values of ERPs, a total sample of 49 people would be needed to detect medium effects (*d* = 0.25) with 91% power using a repeated measure ANOVA (alpha = 0.05) which obtained statistical power at the recommended 0.80 level^125,126^. For behavioral data, to investigate effects of independent variables (task type and judgment type) on behavioral measures, a total sample of 82 people would be needed to detect medium effects (*d* = 0.25) with 89% power using a 2 × 2 ANOVA (alpha = 0.01).

## Data Availability

Raw data associated with any figures can be provided upon request. Further details about the analyses and the data can be requested by contacting the authors.
